# Clinical analysis of lectin-like oxidized low-density lipoprotein receptor-1 in patients with in-stent restenosis after percutaneous coronary intervention

**DOI:** 10.1097/MD.0000000000010366

**Published:** 2018-04-27

**Authors:** Junfeng Liu, Yunde Liu, Kegang Jia, Zhixiao Huo, Qianyu Huo, Zhili Liu, Yongshu Li, Xuejing Han, Rong Wang

**Affiliations:** a Department of Clinical Laboratory, TEDA International Cardiovascular Hospital, Chinese Academy of Medical Science and Beijing Union Medical College; b School of Laboratory Medicine, Tianjin Medical University; c The Second People's Hospital of Tianjin, Tianjin, China.

**Keywords:** in-stent restenosis (ISR), lectin-like oxidized low-density lipoprotein receptor-1 (LOX-1), percutaneous coronary intervention (PCI)

## Abstract

In-stent restenosis (ISR) is the most common complication associated with percutaneous coronary intervention (PCI). Although some studies have reported an association between lectin-like oxidized low-density lipoprotein receptor-1 (LOX-1) and ISR, not enough clinical validation data are available to support this link. Here, we report our cross-sectional study aimed at exploring the feasibility of LOX-1 as a biomarker for the prognostic diagnosis of patients undergoing PCI.

Three groups were included: ISR group, including 99 patients with ISR diagnosed with coronary arteriography (CAG) after PCI; lesion group, comprising 87 patients with coronary artery stenosis (<50%) diagnosed with CAG after PCI; and control group, consisting of 96 volunteers with no coronary artery disease. The levels of LOX-1 were measured in each patient by using an enzyme-linked immunosorbent assay, and their general information as well as laboratory parameters were recorded and followed up during a period of 2 years.

LOX-1 levels gradually increased after PCI along with the progression of the lesion in the 3 groups. The levels of LOX-1 were significantly higher in the ISR group than in the other 2 groups (*P* < .001). LOX-1 levels were correlated with the levels of uric acid (UA) (r = 0.289, *P* = .007), creatinine (CREA) (r = .316, *P* = .003), and high-density lipoprotein cholesterol (HDL-C) (r = −0.271, *P* = .012), whereas no statistically significant correlation was detected with the Gensini score (r = 0.157, *P* = .141). The sensitivity and specificity of LOX-1 were 81.5% and 55.7%, respectively, with the most optimal threshold (5.04 μg/L). The area under curve (AUC) of the receiver operator characteristic (ROC) curve of LOX-1 was 0.720, and LOX-1 had the highest AUC compared with CREA, UA, and HDL-C, both individually and in combination.

A high level of LOX-1 in the early period after PCI has a certain predictive power and diagnostic value for ISR. However, the level of LOX-1 is not related to the Gensini score of coronary artery after PCI, and CREA and UA, which are weakly related to LOX-1, have no obvious synergy in the diagnosis of ISR with LOX-1.

## Abstract 2

Supplemental Digital Content is available in the text

## 1 Introduction

In-stent restenosis (ISR) is the most common complication associated with percutaneous coronary intervention (PCI).^[1]^ Despite the development of balloon angioplasty; bare mental stents (BMSs), which has reduced the rate of coronary stent restenosis from 30%–60% to 16%–44%; and drug-eluting stents (DESs), which has further decreased the coronary stent restenosis rate by up to < 15% depending on the lesion,^[2]^ ISR was still reported to occur in 10% to 30% of patients treated with BMS^[3]^ and in 10.6% of patients treated with sirolimus (rapamycin)-eluting stents.^[4]^ Restenosis after BMS implantation is typically characterized by neointimal hyperplasia (NH) consisting of a proteoglycan matrix and a high proportion of vascular smooth muscle cells (VSMCs). While after DES, restenosis is typically characterized by a proteoglycan-rich NH with relatively few smooth muscle cells.^[5]^ These observations show that NH and remodeling of constricted vasculature, as well as elastic recoil, are predominant causes of arterial restenosis after PCI.^[6]^

NH is an adaptive response of blood vessels to injury and low flow hemodynamic status.^[7,8]^ Many factors (inflammation, thrombosis, VSMC proliferation and migration, extracellular matrix deposition, etc.) cause NH; however, proliferation of VSMCs is considered to become the final common event that is activated by the signaling cascades triggered by these factors. Although DESs inhibit NH to some extent, the drug coating (sirolimus [rapamycin], paclitaxel, etc.) of these devices may suppress the proliferation of VSMCs and thus delay the repair of the vasculature.^[7]^ All currently available DES drugs do not discriminate between proliferating VSMCs and endothelial cells (ECs), the 2 main vascular cell types. The endothelium forms an inner thin layer that serves as an interface between circulating fluid in the lumen and the rest of the vessel wall. The endothelium is crucial owing to its contribution in the regulation of vascular tone, together with its role in suppressing intimal hyperplasia by inhibiting inflammation, thrombus formation, and VSMC proliferation and migration.^[9,10]^ Thus, the endothelium provides a selectively permeable barrier that protects against potentially detrimental circulating factors. Unfortunately, those nonselective DESs not only inhibit VSMCs but also damage ECs, which increases the risk of thrombosis. Hence, finding an effective predictor for the long-term clinical outcome after a successful PCI will benefit both the patient and the hospital.

Lectin-like oxidized low-density lipoprotein (ox-LDL) receptor-1 (LOX-1), the main endothelial receptor for ox-LDL, plays a key role in the genesis and progression of atherosclerosis.^[11]^ LOX-1 is a significant risk factor for atherosclerosis^[12]^ and its levels are increased during hypertension, hyperlipidemia, diabetes, and atheromatous formation,^[12,13]^ as well as in the presence of early and advanced human atherosclerotic plaques, macrophages, and smooth muscle cells.^[14]^ The relationship between LOX-1 and coronary syndrome has also been described.^[15,16]^ However, until now, little validation data have been published to support the translation of evidence on LOX-1 into clinical use. Here, we explore the potential of serum LOX-1 level as a molecular biomarker for the diagnosis of ISR after PCI.

## 2 Materials and methods

### 2.1 Ethics statement

This study was approved by the Ethics Committee of TEDA International Cardiovascular Hospital, in accordance with the Ethical Principles for Biomedical Research Involving Human Subjects (Ministry of Health of the People's Republic of China) and the Declaration of Helsinki for Human Research of 1974 (last modified in 2000). Samples were originally obtained from clinical samples for laboratory diagnoses. After diagnostic testing, excess samples were anonymized and kept for this study. Written informed consent was obtained from each participant (Supplemental Table 1).

### 2.2 PCI

Rapamycin (sirolimus)-eluting stents were used in all patients with PCI, with 1 to 3 stents implanted in coronary artery lesions; cases with only coronary artery balloon dilatation were excluded. All patients undergoing PCI were given aspirin, clopidogrel, and nitrates. Low-molecular heparin calcium, calcium ion antagonists, and beta-receptor blockers were injected subcutaneously, when needed. Patients with hypertension were also given angiotensin-converting enzyme inhibitors or angiotensin receptor blockers; patients with hyperlipidemia were given statins; and patients with diabetes were given antidiabetic drugs or insulin, to maintain blood glucose (Glu) within controllable ranges.

### 2.3 Patient population

From January 2010 to January 2013, 186 patients (from the Department of Cardiology at Tianjin TEDA International Cardiovascular Hospital in China) who had undergone PCI and consented to be followed for 2 years were recruited to participate in this study. These patients were monitored with coronary arteriography (CAG) regularly (every 6 months or more frequently if indicated). CAG was performed by a physician with expertise in heart catheterization, and the final diagnosis was decided by 2 experienced physicians. According to the CAG evaluation, the patients were divided into 2 groups, as described below. A control group comprising persons examined during a regular physical examination was also included.

### 2.4 ISR group

The criterion for inclusion of patients in the ISR group was ≥50% stenosis of the target vessel lumen diameter, as determined with CAG after PCI. The target vessel is the segment of the vessel where the stent was placed, or the 5 mm segment of the vessel on either side of the stent. A total of 99 patients with coronary artery stenosis, who were clinically diagnosed as having acute myocardial infarction (26 cases), unstable angina (50 cases), stable angina (11 cases), obsolete cardiac infarction (9 cases), and exertional angina (3 cases) before PCI, were included in the ISR group. The group included 71 men and 28 women, with an average age of 60.37 ± 8.98 years.

### 2.5 Non-significant lesion group

Eighty-seven patients with coronary artery lumen diameter stenosis < 50% diagnosed with CAG after PCI, and clinically diagnosed as having acute myocardial infarction (27 cases), unstable angina (31 cases), stable angina (14 cases), exertional angina (8 cases), obsolete cardiac infarction (6 cases), and asymptomatic myocardial ischemia (1 case) before PCI, were included in the non-significant lesion group, which included 57 men and 30 women, with an average age of 59.09 ± 9.19 years.

### 2.6 Control group

Ninety-six volunteers with no coronary artery syndrome were randomly recruited from persons given regular physical examinations at our hospital. These volunteers served as the control group, comprising 60 men and 36 women, with an average age of 60.06 ± 9.49 years.

The exclusion criteria for individuals in all 3 groups were deep vein thrombosis, cerebral embolism, hemolytic disease, pulmonary embolism, disseminated intravascular coagulation, and other diseases. Factors associated with the ISR after PCI, including post-ISR medication, hypertension, smoking, diabetes, body mass index (BMI), and cardiac function, were summarized.

### 2.7 Specimen collection and detection

Blood samples were taken from an antecubital vein in all participants, after >12 hours of fasting after angiography. Total 7.7 mL blood were collected from patient and distributed in 3 tubes. Among them, a separation gel tube with 3 mL blood was centrifuged at relative centrifugal force 1408 gravity for10 minutes. LOX-1and other biochemical tests were measured with the separated serum. LOX-1 was detected by enzyme linked immunosorbent assay with a 550 enzyme microplate reader (American Bio-Rad Co, Hercules, CA). Reagents were purchased from Hermes Criterion Biotechnology (HCB, Vancouver, Canada). Optical density values of the samples were converted to the concentration values of LOX-1 according to the standard curve drawn by the 4-parameter logistic curve fitting. Total cholesterol (TC), triglyceride (TG), high-density lipoprotein cholesterol (HDL-C), low-density lipoprotein cholesterol (LDL-C), uric acid (UA), creatinine (CREA), Glu, aspartate aminotransferase mitochondrial isoenzyme (ASTm), and creatine kinase MB isoenzyme (CK-MB) were measured with a Hitachi 7600 automated biochemical analyzer (Hitachi, Tokyo, Japan). The levels of TC, TG, HDL-C, LDL-C, UA, CREA, and Glu were measured using Wako reagents (Wako Pure Chemicals Co, Tokyo, Japan). Detection reagents for CK-MB were obtained from Beijing Strong Biotechnologies Inc. (Beijing, China). Detection reagents for ASTm were from Shanghai Beijia Company (Shanghai, China). The estimated glomerular filtration rate (eGFR) was calculated as 175 × (CREA/88.4)^−1.154^ × (age)^−0.203^ for men and 175 × (CREA / 88.4)^−1.154^ × (age)^−0.203^ × 0.742 for women.^[17]^

Another 2.7 mL blood was collected with a sodium citrate anticoagulant tube, which centrifuged at 1408 g for10 minutes to determinate the level of Fibrinogen and D-dimer. Fibrinogen and D-dimer was measured with a Sysmex CS2000i coagulation analyzer (Sysmex, Kobe, Japan) and using Siemens reagents (Siemens, Marburg, Germany).

The last 2 mL blood was collected with ethylene diamine tetracetic acid-K2 anticoagulant tube for the determination of platelet count (PLT), mean platelet volume (MPV), platelet volume distribution width (PDW), platelet large cell ratio (PLCR), and plateletcrit (PCT). PLT, MPV, PDW, PLCR, and PCT were detected with a Sysmex XE2100 blood analyzer using Sysmex reagents produced in Japan.

### 2.8 Gensini score

The Gensini score was evaluated according to the method by Gensini.^[18]^ Briefly, all coronary artery lesions were evaluated quantitatively. Gensini scores were defined according to the degree of coronary artery lumen stenosis, as follows: 1 point (0%–25%), 2 points (25%–50%), 4 points (51%–75%), 8 points (76%–90%), 16 points (91%–99%), and 32 points (100%). The score is equal to the degree of coronary artery stenosis multiplied by the weight coefficient of the lesion vessel. The final score for each case in the ISR group and the non-significant lesion group was the sum of the scores for all branches.

### 2.9 Statistical analysis

All data were analyzed with SPSS19.0 software (SPSS Inc, Chicago, IL). Measurement data with a normal distribution were expressed as mean ± standard deviation. Parameters were compared among the 3 groups by using 1-way analysis of variance, followed by the least significant difference-t test between the 2 groups. Measurement data that did not have a normal distribution after natural logarithm transformation were expressed as median, M (P25, P75). Parameters were compared among the 3 groups by using Kruskal–Wallis H analysis, followed by the Nemenyi test for comparisons between 2 groups. Enumeration data were expressed as percentage (%) and were determined with the *χ*^2^ test. Spearman correlation analysis was carried out among LOX-1, Gensini scores, and other variables. Receiver operating characteristic (ROC) curve analysis was used for ISR after PCI. Nonconditional multivariate logistic regression analysis was performed using the statistically significant variables through a 3-group comparison. *P* values < .05 were considered difference, and *P* values < .001 were considered significant difference.

## 3 Results

### 3.1 Analysis of clinical characteristics of the 3 groups

The clinical characteristics of the patients and controls included in this study are listed in Table 1, which shows no significant differences (*P* > .05) among the 3 groups in age, sex, BMI, hypertension (yes/no), diabetes mellitus (yes/no), TC, LDL-C, MPV, PDW, PLCR, eGFR, left ventricular ejection fraction, left ventricular diastolic diameter, left ventricular posterior wall, interventricular septum thickness, and left ventricular end-systolic dimension (Table 1).

**Table 1:**
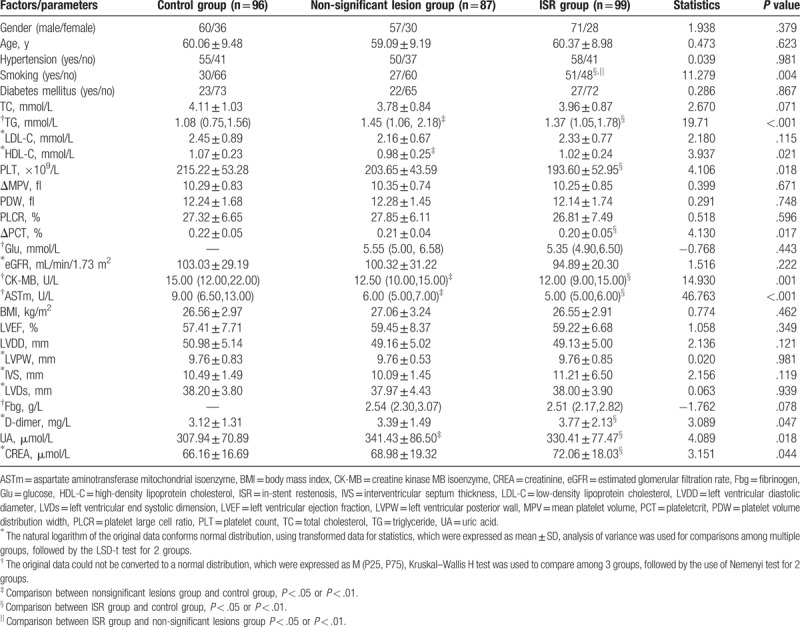
The clinical characteristics in 3 groups.

Smoking (yes/no), TG, HDL-C, PLT, PCT, CK-MB, ASTm, D-dimer, UA, and CREA were significantly different among the 3 groups (*P* < .05, Table 1). The ISR group and the control group were significantly different (*P* < .05) in terms of smoking (yes/no), TG, PLT, PCT, CK-MB, ASTm, D-dimer, UA, and CREA, whereas the non-significant lesion group and the control group were significantly different (*P* < .01) with regard to TG, HDL-C, CK-MB, ASTm, and UA (Table 1).

### 3.2 Level of LOX-1 is associated with ISR progression after PCI

**Figure 1:**
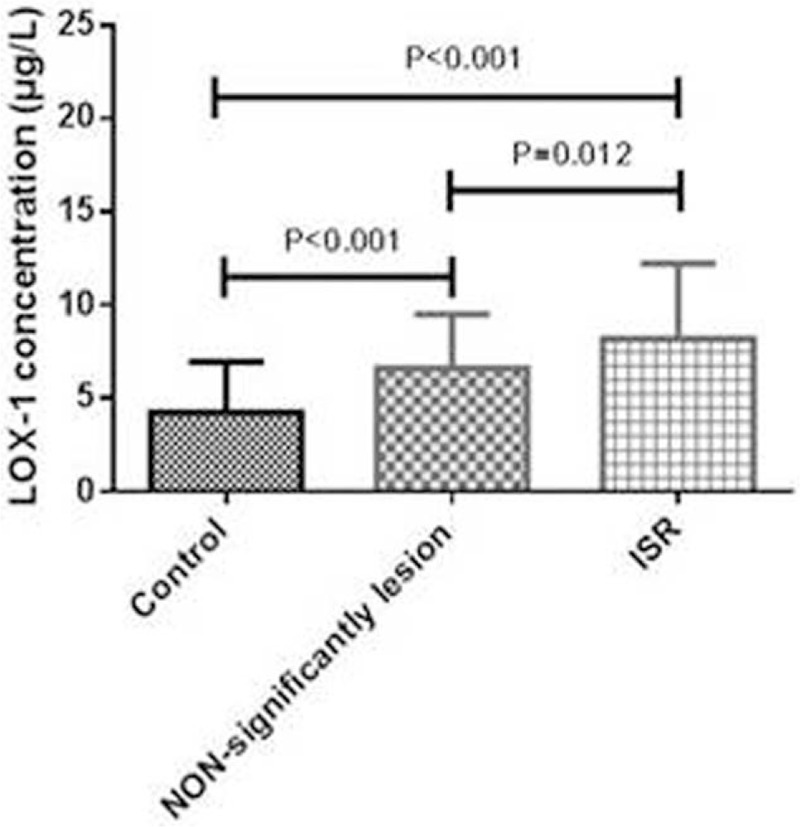
The levels of LOX-1 among the 3 groups. ISR = in-stent restenosis, LOX-1 = lectin-like oxidized low-density lipoprotein receptor-1. The original data could not be converted to a normal distribution, which were expressed as M(P25, P75). Kruskal–Wallis H test was used to compare among 3 groups, followed by using Nemenyi test for between 2 groups. Comparison between nonsignificant lesions group and control group, *P* < .001. Comparison between ISR group and control group, *P* < .001. Comparison between ISR group and nonsignificant lesions group, *P* =.012.

The level of LOX-1 was strongly associated with the progression of ISR (r = 0.448, *P* < .001). As shown in Fig. 1, the LOX-1 levels were the highest (H = 64.942, *P* < .001) in patients with ISR, and the difference was significant compared with that in patients with nonsignificant lesions (*P* = .012) and that in the control group (*P* < .001). In fact, the LOX-1 levels in both patient groups (ISR and non-significant lesion groups) were significantly higher than that in the control group (*P* < .001).

**Figure 2:**
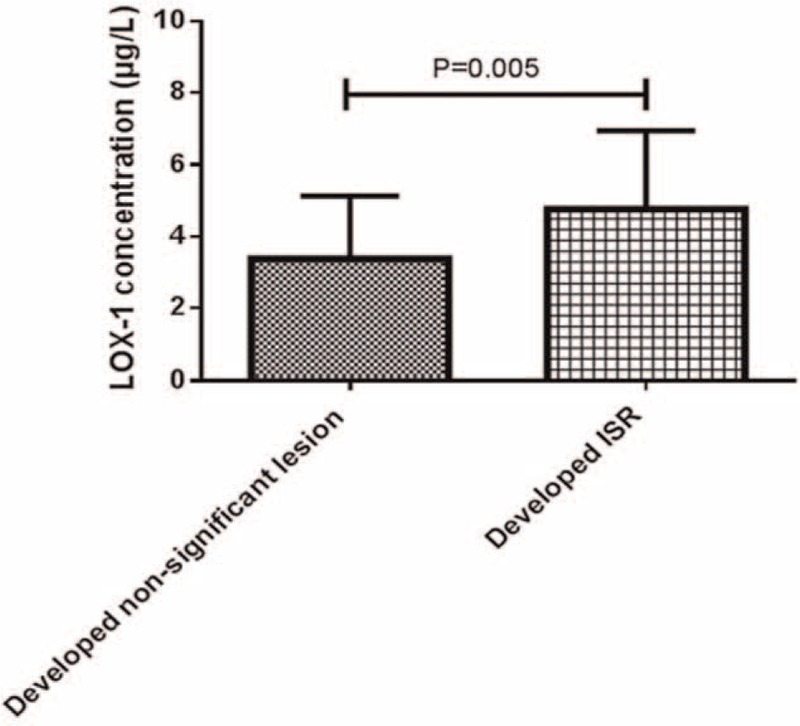
The level of LOX-1between ISR and nonsignificant lesions early after PCI. ISR = in-stent restenosis, LOX-1 = lectin-like oxidized low-density lipoprotein receptor-1. The original data could not be converted to a normal distribution, which were expressed as M(P25, P75). Mann–Whitney *U* test was applied to compare between developed nonsignificant lesion and developed ISR (*P* = .005).

The LOX-1 levels during the early post-PCI period (1–7 days) were compared between 41 patients who developed ISR and 51 patients developed with nonsignificant lesions. Figure 2 shows that the medium levels of LOX-1 in the ISR group were significantly higher than the LOX-1 levels in the nonsignificant lesion group (Z = 2.781, *P* = .005), suggesting that the higher level of LOX-1 during the early post-PCI period is predictive of ISR.

### 3.3 LOX-1 is an independent risk factor after PCI

The Gensini score is used to evaluate the degree of coronary artery stenosis. In the ISR group, Spearman correlation analysis showed no statistically significant correlation between the concentrations of LOX-1 (r = 0.157, *P* = .141) and the Gensini score. However, LOX-1 had a marginal correlation with UA (r = 0.289, *P* = .007), CREA (r = 0.316, *P* = .003), and HDL-C (r = −0.271, *P* = .012), but there was no collinearity between them (Table 2).

**Table 2:**
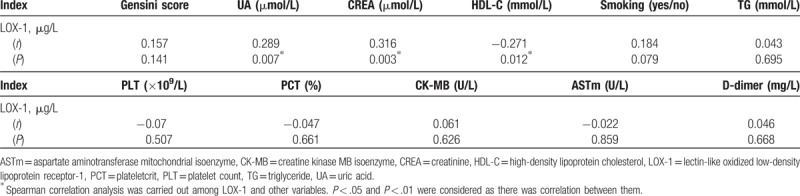
Correlation analysis between LOX-1and Gensini scores and significant variables.

Subsequently, we conducted nonconditional multivariate logistic regression analysis by using the statistically significant variables (smoking, TG, PLT, PCT, CK-MB, ASTm, LOX-1, D-dimer, UA, CREA, and HDL-C) as independent variables, and if restenosis occurred (yes [restenosis]: 1, no [control]: 0) as dependent variables. The results showed that LOX-1 (odds ratio = 1.803, 95% confidence interval = 1.250–2.601, *P* = .002) is an independent risk factor for ISR after PCI (Table 3).

**Table 3:**
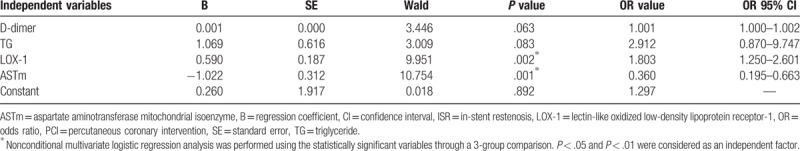
Multiple factors logistic regression for ISR after PCI.

### 3.4 Clinical evaluation indices of LOX-1 for the diagnosis of ISR

**Figure 3:**
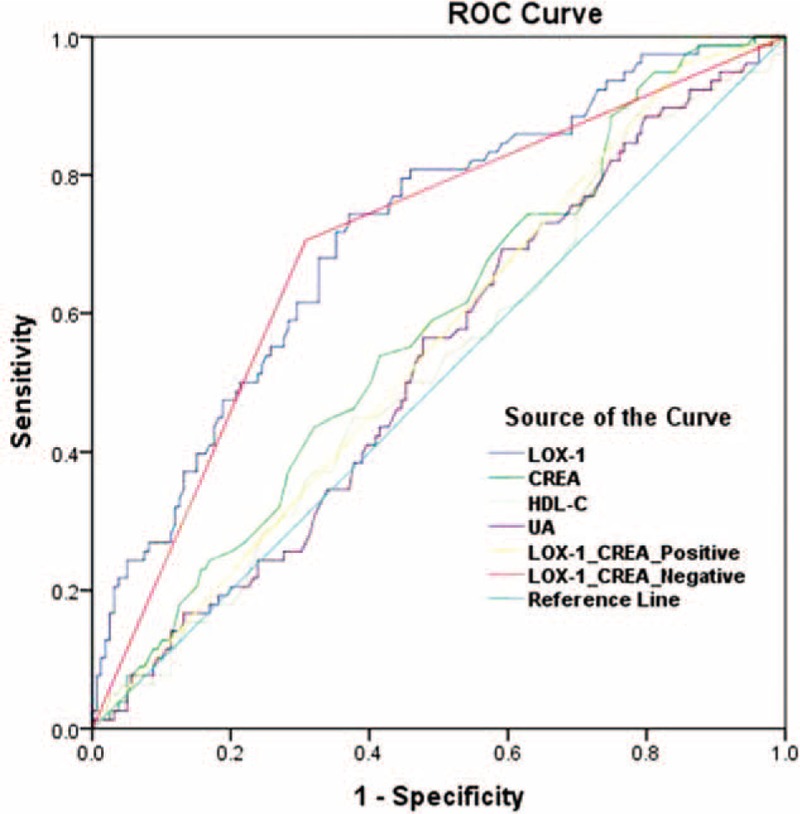
The ROC curve of LOX-1, CREA, UA, HDL-Cand combinations. CREA = creatinine, HDL-C = high-density lipoprotein cholesterol, LOX-1_CREA_Negative = combination the negative results of both LOX-1+CREA, LOX-1_CREA_Positive = combination the positive results of both LOX-1+CREA, LOX-1 = lectin-like oxidized low-density lipoprotein receptor-1, ROC = receiver operator characteristic, UA = uric acid.

As LOX-1 showed a correlation with UA, CREA, and HDL-C, we combined these factors with LOX-1 for further clinical analysis. The ROC curve in Fig. 3 demonstrates that the AUC of LOX-1 was 0.720, ranked the highest; this represents the best value for clinical diagnosis, compared with both CREA individually and in combination. Table 3 lists the sensitivity and specificity of LOX-1 as 81.5% and 55.7%, respectively, with the most optimal threshold (5.04 μg/L). Both the positive predictive value (PPV) (48.1%) and negative predictive value (NPV) (85.7%) of LOX-1 were much better than the other associated markers. CREA had a higher sensitivity (93%) than LOX-1 (81.5%); however, the specificity of CREA (20.9%) was low, which resulted in a lower PPV (38.5%). UA (*P* = .65) and HDL-C (*P* *=* .616) has no diagnosis power, since both were not significantly different between the ISR and non-ISR groups (non-significant lesion and control groups) (Table 4).

**Table 4:**
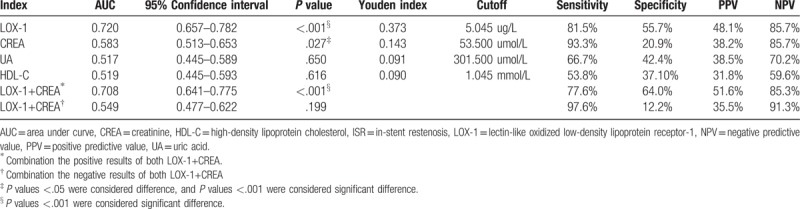
The clinical evaluation of LOX-1 for the diagnosis of ISR group.

Additionally, the combination of the positive results of LOX-1 and CREA yielded no improvements in terms of sensitivity, nor did combining their negative results enhanced specificity (Table 4).

## 4 Discussion

Current medical treatments decrease but do not prevent the development of atherosclerosis. PCI is an important method of treating coronary artery stenosis. Percutaneous transluminal coronary angioplasty (PTCA) was first used in 1977, and has evolved greatly from the early days of using a stent balloon to the present stage. In the era balloon dilations, the restenosis rate of target vessels was 32% to 40%, assessed 6 months after PTCA. In the BMS era, the restenosis rate was as high as 17% to 32%. Finally, in the current DES era, the restenosis rate was reported to be about 10%.^[19]^

Rapamycin-eluting stents, which are used in treating atherosclerotic blockages in coronary arteries, were approved by the Food and Drug Administration in 2003 for use in preventing ISR.^[20]^ Concerning the mechanism of action of rapamycin, an inhibitor of mammalian target of rapamycin (mTOR) phosphorylation, studies have demonstrated that rapamycin inhibits VSMC proliferation and migration through the upregulation of the cell-cycle regulator p27Kip1, inhibiting the G1-to-S phase transition of cells and ultimately leading to cell growth arrest. Recently, Zhou et al^[21]^ reported that rapamycin inhibits ox-LOX uptake in human umbilical vein ECs via the mTOR/nuclear factor-κB / LOX-1 pathway.

LOX-1 is a 52-kDa surface receptor that is a type II membrane protein belonging to the C-type lectin family.^[22]^ LOX-1 is an ox-LDL receptor in ECs.^[11]^ In normal conditions, the expression of LOX-1 is very low; however, in pathological conditions such as hypertension, diabetes, myocardial ischemia, hyperlipidemia, ischemic reperfusion injury, transplantation, and atherosclerosis, LOX-1 expression is upregulated.^[23]^ Previous literature supports the presence of an association between LOX-1 and ISR. LOX-1 is involved in the pathophysiology of atherosclerosis;^[24]^ it may mediate endocytosis of ox-LDL by the independent grid protein internalization pathway, which can maximally combine with ox-LDL, thereby increasing vascular endothelial dysfunction and atherosclerosis.^[25]^

In addition, proliferation and migration of VSMCs play key roles in vascular NH after PCI, which is a crucial factor leading to restenosis.^[8,26–28]^ Furthermore, the molecular mechanisms of LOX-1 expression or mediated signal transduction system are partially similar to the signaling systems of VSMC proliferation and migration.^[26,27,29,30]^ For instance, inhibition of protein kinase C can significantly reduce the expression of LOX-1, and inhibit intimal proliferation and migration of VSMC, thus preventing restenosis.^[26,31]^ This shows that LOX-1 plays a catalytic role in the occurrence and development of restenosis after PCI. Finally, LOX-1 is believed to be a predictor of coronary heart diseases and cardiovascular events.^[32]^ In stable coronary artery disease, patients with lesions of the proximal/middle segment of left anterior descending (LAD) coronary artery have significantly higher circulating soluble LOX-1 levels than patients with lesions in the distal segments of the LAD; thus, LOX-1 levels are associated with the proximal/middle segment of LAD lesions.^[33]^

Consistent with the above findings, our data show that LOX-1 levels are associated with the PCI procedure (r = 0.4448, *P* < .001), and that patients in the ISR group had significantly increased levels of circulating LOX-1, especially during the early stage after PCI. We also documented that the levels of LOX-1 were not correlated with the Gensini score. Together, these findings suggest that LOX-1 is a promising predictive biomarker for ISR. It is involved in the process of restenosis after PCI but has no relationship to the degree of restenosis.

Spearman correlation analysis in the ISR group showed a positive correlation between LOX-1 and UA (r = 0.289, *P* = .007) as well as CREA (r = 0.316, *P* = 0.003) but no collinearity. This phenomenon may reflect the relationship between decreased renal function and cardiovascular disease. Coronary artery stenosis affects the patient's heart functions, which results in different degrees of loss of cardiac functions: renal hypovolemia and reduced perfusion are major causes of impaired renal function, and lead to increases in the levels of UA and CREA. Based on our results, we conclude that LOX-1 can also be identified as an independent risk factor for ISR, as evidenced by both multivariate logistic regression analysis and ROC curve analysis.

The ROC curve is a fundamental tool for diagnostic test evaluation. To the best of our knowledge, this is the first study to perform ROC clinical analysis to evaluate the possibility of LOX-1 as a biomarker. In Fig. 3, the ROC curve and AUC show that LOX-1 (0.720) ranks the highest compared with others, when considered individually or in combination. Although CREA had a higher sensitivity (93.3%) than LOX-1 (81.5%), the specificity of LOX-1 (55.7%) was much higher than that of CREA (20.9%). Furthermore, combining LOX-1 and CREA did not result in considerable improvement. We did not combine LOX-1 neither with UA nor with HDL-C because the AUC calculation showed that UA and HDL-C was not significantly different between the ISR and non-ISR (nonsignificant lesion and control) groups. We also analyzed the AUC of LOX-1 in the nonsignificant lesion group but saw no significant differences as well (data not shown). These results show that the level of LOX-1 has a predictive power for ISR during the post-PCI period, and support our conclusion that LOX-1 is also an independent risk factor for ISR. As a rapidly highly expressed or overexpressed gene induced by mechanical stimulation, LOX-1 might be significantly expressed in ECs and VSMCs in early NH after PCI. As ox-LDL plays a key role in the pathogenesis of atherosclerosis, selective inhibition of the intake of ox-LDL by ECs is a promising therapeutic approach. LOX-1 is one of the most important target molecules in ECs, and the combination of restriction between LOX-1 and ox-LDL can be a new target for preventing endothelial dysfunction and ISR.^[12,34]^

In conclusion, a high level of LOX-1 in the early period after PCI has a certain predictive power and diagnostic value for ISR. However, the level of LOX-1 is not related to the Gensini score of coronary artery after PCI, and CREA and UA, which are weakly related to LOX-1, have no obvious synergy in the diagnosis of ISR with LOX-1. Owing to the relatively lower PPV of LOX-1, it is better to combine LOX-1 with other biomarkers to enhance the specificity. Summarize, there are several strengths to convince our conclusions including add the control group as the baseline, increase the follow-up time to 2 years to decrease the effect induced by some antiplatelet agents after PCI etc. However, the limitations should be addressed as well, for instance, the number of enrolled patients should be increased, and the differences of LOX-1 in terms of ethnicity, sex, and specific cardiovascular diseases, which may influence the overall conclusion, should be investigated.

In addition, ISR after rapamycin-eluting stents is a chronic and complex process, oxidative stress and chronic inflammation play a promoting role in the formation of in-stent neoatherosclerosis^[35]^ Currently, non-coding RNAs and microRNAs are reported to show a prognostic potential to predict future ISR after PCI.^[36]^ Among them, the miR-133 is more attractively.^[37]^ However, microRNA biomarkers are difficult to enter to clinical practice since they are labor-consuming and lower specificity. Other promising potential biomarkers including inflammatory molecules, or those related to the oxidative stress should be sustainable identified.^[35]^

## Author contributions

**Conceptualization:** Junfeng Liu, Yunde Liu, Kegang Jia.

**Data curation:** Junfeng Liu, Kegang Jia, Yong shu Li, Xuejing Han.

**Formal analysis:** Junfeng Liu, Zhixiao Huo, Rong Wang.

**Funding acquisition:** Junfeng Liu, Rong Wang.

**Investigation:** Junfeng Liu, Kegang Jia.

**Methodology:** Junfeng Liu, Qianyu Huo.

**Project administration:** Junfeng Liu, Rong Wang.

**Resources:** Junfeng Liu, Yunde Liu, Kegang Jia.

**Software:** Zhixiao Huo, Xuejing Han.

**Supervision:** Yunde Liu, Kegang Jia, Rong Wang.

**Validation:** Junfeng Liu, Qianyu Huo, Zhili Liu, Yongshu Li, Xuejing Han.

**Writing – original draft:** Junfeng Liu, Zhixiao Huo, Rong Wang.

**Writing – review & editing:** Rong Wang.

## Supplementary Material

Supplemental Digital Content
