# Supplementary material for: Clinical analysis of lectin-like oxidized low-density lipoprotein receptor-1 in patients with in-stent restenosis after percutaneous coronary intervention
Source: Medicine (Baltimore). 2018 Apr 27;97(17):e0366. doi: 10.1097/MD.0000000000010366 (PMC5944531; doi:10.1097/MD.0000000000010366)
Supplement: Supplemental Digital Content [file medi-97-e0366-s001.docx]

Supplementary Table 1

**Informed Consent**

**（**Notification of illness within 24 hours of admission**）**

Patient and his/her families confirm the medical history information provided：

Patient name**：** Gender**：** Age**：**

The medical history data of in hospital records**：**

Preliminary assessment notification

1. At present the initial diagnosis**：**

**2.** At present further diagnosis and treatment plan are needed**：**

**3.** Current the patient’s clinical characteristics and matters need attention**：**

**4.** According to standardized management requirements, the patient suffering from diseases may be bring into related clinical pathways and single disease management in our hospital.

**5.**  The patient's medical records, imaging data and biological samples can used in medical research during the current admission, we promise to strictly comply with the relevant norms and will not disclose patient’s privacy.

Medical staff in our department wholeheartedly service for patient who in the hospitalization period in our hospital based on people-oriented/curing the sickness to save patient. If you have any problems related to the disease, please contact your doctor promptly.

The doctor's advice is known. The medical history information on patient has been confirmed and accurate.

Signature of patient / Client / Guardian： Relationship with patients：

Evaluation and notification physician signature：
